# Short-term perceptual reweighting in suprasegmental categorization

**DOI:** 10.3758/s13423-022-02146-5

**Published:** 2022-08-01

**Authors:** Kyle Jasmin, Adam Tierney, Chisom Obasih, Lori Holt

**Affiliations:** 1 Department of Psychology, Wolfson Building, Royal Holloway, University of London, Egham, Surrey TW20 0EX UK; 2 Birkbeck, University of London, London, UK; 3 Carnegie Mellon University, Pittsburgh, PA USA

**Keywords:** Speech, Prosody, Dimension-based statistical learning, Suprasegmental speech, Perceptual cue weighting

## Abstract

Segmental speech units such as phonemes are described as multidimensional categories whose perception involves contributions from multiple acoustic input dimensions, and the relative perceptual weights of these dimensions respond dynamically to context. For example, when speech is altered to create an “accent” in which two acoustic dimensions are correlated in a manner opposite that of long-term experience, the dimension that carries less perceptual weight is down-weighted to contribute less in category decisions. It remains unclear, however, whether this short-term reweighting extends to perception of suprasegmental features that span multiple phonemes, syllables, or words, in part because it has remained debatable whether suprasegmental features are perceived categorically. Here, we investigated the relative contribution of two acoustic dimensions to word emphasis. Participants categorized instances of a two-word phrase pronounced with typical covariation of fundamental frequency (F0) and duration, and in the context of an artificial “accent” in which F0 and duration (established in prior research on English speech as “primary” and “secondary” dimensions, respectively) covaried atypically. When categorizing “accented” speech, listeners rapidly down-weighted the secondary dimension (duration). This result indicates that listeners continually track short-term regularities across speech input and dynamically adjust the weight of acoustic evidence for suprasegmental decisions. Thus, dimension-based statistical learning appears to be a widespread phenomenon in speech perception extending to both segmental and suprasegmental categorization.

**Supplementary Information:**

The online version contains supplementary material available at 10.3758/s13423-022-02146-5.

A central challenge in the study of speech communication is understanding how continuous variation across multiple acoustic dimensions is mapped onto linguistic representations. Segmental speech categories such as phonemes are not signalled by any single acoustic dimension. Instead, phonemes are conveyed by multiple acoustic dimensions that vary in their diagnosticity or “perceptual weight” in signalling a speech category (Holt & Lotto, 2006; Toscano & McMurray, 2010). For example, in clear speech, the phoneme /b/ (as in “bat”) is distinguished from /p/ (as in “pat”) in part by the time elapsed between the acoustic burst created by release of the articulators and the onset of the periodic signal associated with the vibration of the vocal folds, an interval referred to as the “voice onset time,” or VOT (Lisker, 1957), which is longer for /p/ than /b/. Whereas VOT is the most reliable cue signalling /b/–/p/ categorization in clear speech for English listeners, at least 16 other acoustic dimensions also contribute, such as the fundamental frequency (F0) at VOT offset and the length of delay in the onset of the first formant (Lisker, 1986). Thus, multiple acoustic dimensions contribute to segmental speech categorization, but the diagnosticity of these dimensions in signalling segmental speech categories varies: dimensions carry different *perceptual weight*.

Perceptual weights of acoustic dimensions are context-dependent: When listeners encounter short-term changes in the ways in which dimensions are associated with categories, perceptual weights adjust in response. In particular, perceptual weights of acoustic dimensions rapidly shift in response to short-term changes in the distribution of acoustic cues experienced in speech input, such as when encountering a talker with an accent. For example, in English, VOT and F0 typically covary such that longer VOT and higher F0 co-occur and signal /p/, whereas shorter VOT and lower F0 frequencies co-occur and signal /b/. When listeners are exposed to an artificial “accent” for which the relationship between F0 and VOT is reversed (e.g., longer VOTs co-occurring with lower F0), they rapidly down-weight reliance on the secondary dimension such that F0 is no longer an effective signal of /b/ versus /p/ category membership (Idemaru & Holt, 2011, 2014, 2020; Lehet & Holt, 2017; Schertz et al., 2016; Wu & Holt, 2022; Zhang & Holt, 2018). When the short-term input regularities return to English norms, the perceptual weight of F0 quickly returns to baseline levels such that it signals /b/ and /p/ differentially. Importantly, several lines of evidence (Idemaru & Holt, 2011; R. Liu & Holt, 2015; Wu & Holt, 2022; Zhang et al., 2021) demonstrate that activation of an existing categorical linguistic representation is crucial to eliciting dynamic reweighting of secondary acoustic input dimensions. This suggests that down-weighting of a particular dimension occurs when a categorical representation is activated but the value of that dimension does not match the range of values normally associated with that category, with short-term weight adjustment occurring via supervised error-driven learning (Guediche et al., 2014; Wu & Holt, 2022) or, alternatively, reinforcement learning mechanisms (Harmon et al., 2019).

It is not yet known whether short-term reweighting of acoustic dimensions extends beyond segmental perception to suprasegmental aspects of speech perception that span multiple phonemes, such as lexical stress, emphasis, and phrase boundaries. Like segmental features, suprasegmental features are correlated with variation in multiple acoustic dimensions, and some of these dimensions more reliably signal the presence of a suprasegmental feature and are weighted more highly by listeners. For example, emphasized words have higher F0, longer duration, and greater amplitude in English (Breen et al., 2010). F0 is the most consistent cue and is accordingly weighted most highly by listeners on average (Jasmin, Dick, Holt, & Tierney, 2020a). One might predict, therefore, that if listeners were exposed to an artificial “accent” in which F0 and duration were correlated in a manner opposite to English expectations, that duration would be down-weighted, due to it being a secondary cue. However, as explained above, this prediction is dependent on listeners activating a multidimensional categorical linguistic representation of word emphasis, and it remains under debate whether perception of suprasegmental features involves the activation of discrete multidimensional categories. (Here we define “suprasegmental category” as a learned linguistic representation, cued by variation in multiple acoustic dimensions spanning multiple phonemes, that is discrete inasmuch as there is a sharp boundary in multidimensional acoustic space where perception rapidly shifts from hearing one category to hearing another. Nevertheless, this definition allows the possibility that acoustic variation within a category space can modulate the strength of perception of the category.) While some linguistic theories of suprasegmental features posit the existence of discrete categories (Pierrehumbert & Hirschberg, 1990; Xu & Xu, 2005), others suggest that suprasegmental information is encoded in a graded and dimension-specific fashion (Aylett & Turk, 2004).

Prior experimental psycholinguistic research has sought to investigate whether suprasegmental features are perceived as graded or discrete. Two main methodologies have been used: categorical perception and imitation. In the classic categorical perception paradigm, a peak in discriminability that aligns with a categorization boundary is taken as evidence for categorical perception. Evidence for categorical perception of suprasegmentals has been mixed. It has been reported for F0 peak alignment (Kohler, 1987) and high versus low boundary tones (Remijsen & van Heuven, 1999; Saindon et al., 2017a, b; Schneider & Lintfert, 2003). However, other papers have reported finding no discrimination peak for high versus low boundary tones (Falé & Faria, 2006) or emphatic (vs. neutral) accents (Ladd & Morton, 1997). Kimball and Cole (2020) manipulated the extent to which both F0 and duration implied the existence of an accent on an earlier versus later word in a phrase, and then compared the degree of categorical perception to that found for a fricative contrast. Although a discrimination peak was clearly present for fricative perception, there was no evidence for a discrimination peak for accent perception. Results from imitation paradigms have sometimes supported the existence of pitch-based prosodic categories, with clear evidence for minimized within-category differences in imitation of F0 peak alignment (Pierrehumbert & Steele, 1989; Zárate-Sández, 2016) and high versus low boundary tones (Braun et al., 2006). However, other studies have demonstrated graded imitations of stimuli differing in type of pitch accent (Dilley, 2010) and have provided evidence that while one pitch-based cue (accent down-step) is perceived categorically, several others (duration, peak height, and peak alignment) are perceived as gradients (Baumann et al., 2006).

Despite prior research, therefore, it remains an open question whether listeners activate multidimensional discrete categories when perceiving suprasegmental features such as word emphasis. One way to test the hypothesis that listeners perceive word emphasis as a multidimensional discrete suprasegmental category is to expose participants to an “accent” that reverses the typical relationship between primary (F0) and secondary (duration) dimensions associated with word emphasis. If a multidimensional category is activated during accent exposure, then its activation will not be consistent with the value along the secondary dimension, and duration will be subsequently down-weighted as a cue to word emphasis. If, on the other hand, no such multidimensional category is activated, and F0 and duration are evaluated by listeners independently, then reversing the typical relationship between F0 and duration dimensions should have no effect on subsequent duration weighting. Here, to adjudicate between these two possibilities, we presented participants with spoken phrases drawn from a two-dimensional stimulus space in which stimuli varied in the extent to which F0 contours and duration patterns implied emphasis on one of two words.

## Methods

### Participants

Native monolingual speakers of American English (*N* = 43, 37 females; ages 18–22 years) with normal hearing were recruited from Carnegie Mellon University. Participants took part for university credit or payment after giving informed consent. The study was approved by the Carnegie Mellon University Institutional Review Board in line with the Declaration of Helsinki. Because dimension-based statistical learning paradigms had not been used previously to examine a suprasegmental contrast, the final sample size was determined by collecting the maximum number of participants that could be recruited and tested given time and resource constraints.

### Stimulus creation

The stimulus space was defined by orthogonal acoustic manipulations across duration and F0 contour over tokens of the spoken English phrase “study music.” The tokens were created by recording the voice of a native English speaker speaking the phrases “Dave likes to STUDY music” (early focus) and “Dave likes to study MUSIC” (late focus), with emphasis placed either on STUDY, or MUSIC. The two recordings were then “morphed” together using STRAIGHT software (Jasmin et al., 2020a, b, c; Jasmin et al., 2021; Kawahara & Irino, 2005): The F0 was extracted from voiced segments of the two utterances; next, aperiodic aspects of the signal were identified and analyzed; then, the filter characteristics of the signal were calculated. Finally, the two “morphing substrates” (speech from each recording decomposed into F0, aperiodic aspects, and filter characteristics) were manually time aligned by marking corresponding “anchor points” in both recordings. This was done by examining a similarity matrix generated by STRAIGHT (based on the two input sound files) and manually marking corresponding salient changes in the spectrograms. For full details see Online Supplemental Materials and Jasmin et al., 2021.

Following temporal alignment, STRAIGHT’s morphing procedure involves regenerating a signal using a linear interpolation between the manually-marked anchor points in an abstract distance space (Kawahara & Irino, 2005). For F0 this is in the log-frequency domain. In creating these morphed versions, the F0 contour and durational morphing rates were adjusted orthogonally in order to create a 7 × 7 grid of stimuli whose F0 and durational properties cued emphasis on STUDY or on MUSIC to seven different degrees: 0%, 17%, 33%, 50%, 67%, 83%, and 100%, with 0% indicating that the F0 contour or duration characteristics came from the “STUDY music” recording, 100% meaning F0 and duration were identical to the “study MUSIC” recording, and intermediate values indicating F0 and duration patterns linearly interpolated between the two original recordings. All other acoustic cues, including amplitude, were equated across the two examples as a part of the selective morphing process. Finally, the stimuli were trimmed to only contain the two words “study” and “music.” Following morphing, the differences in F0 between study and music, measured at the nucleus of the first vowel of each word, at each of the seven F0 levels were −8.5, −5.0, −2.1, +0.6, +3.4 +5.7, and +8.1 semitones, negative values reflecting higher frequency F0 on “music” than “study.” (These steps were not exactly evenly spaced as they reflect the difference in measurements between the two words for F0.) The differences in duration between “study” and “music” in the final morphed stimuli were approximately 0.12, 0.08, 0.05, 0.02, −0.02, and −0.06, and −0.08 seconds. Plots of F0 and Duration values for each stimulus level, as well as graphical depictions of the stimulus level steps, are found in the Online Supplemental Materials.

### Baseline stimuli

**Fig. 1:**
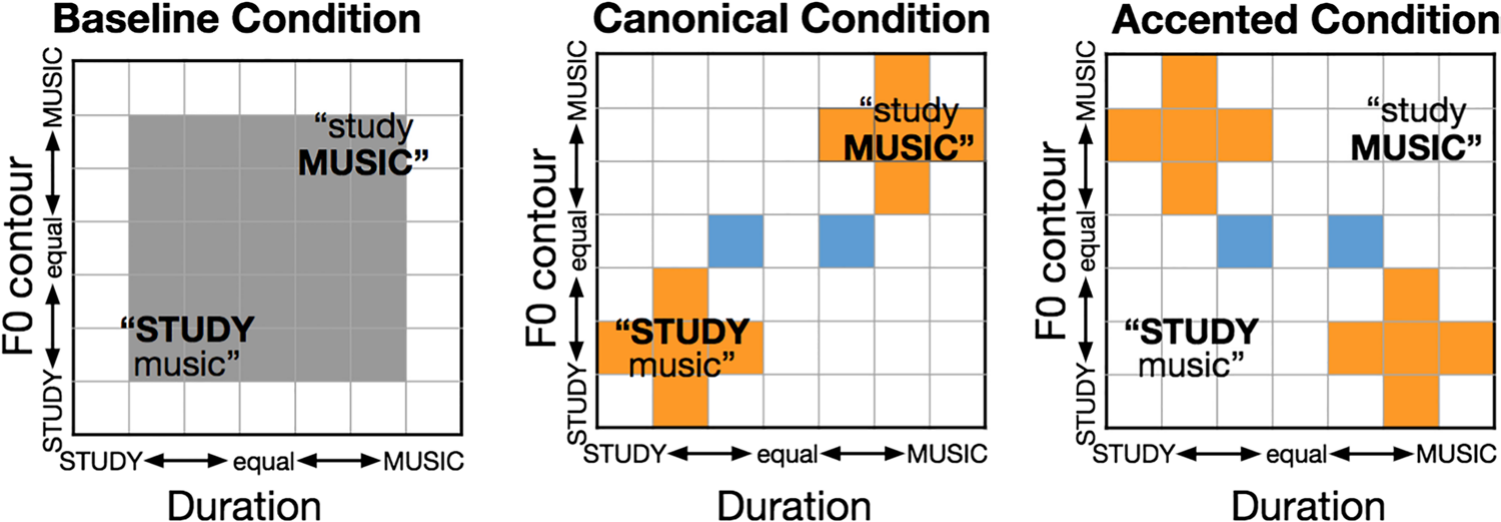
Stimuli. Stimuli sampled a 7 × 7 acoustic space across duration and F0 contour. Baseline categorization measurements made use of the center 25 stimuli in the grid (left panel), sampled orthogonally across dimensions. During the canonical block (middle panel), participants categorized canonical-exposure stimuli (orange squares). During accented exposure (right panel), participants categorized stimuli for which F0 and duration cues possessed a correlation opposite that of English (orange squares). Participants also categorized *test* stimuli, which had identical F0 contours but distinct durations (blue squares). (Color figure online)

Figure 1 illustrates how stimuli were sampled from this 7 × 7 stimulus space across blocks. Baseline stimuli consisted of 25 versions of the spoken phrase “study music” with word emphasis manipulated across F0 contour and duration. A 5 × 5 subset from the center of the 7 × 7 stimulus space (grey in Fig. 1) sampled the two acoustic dimensions orthogonally to establish listeners’ baseline perceptual weights in labeling the speech as having early versus late word emphasis (*STUDY music* versus *study MUSIC*).

### Exposure stimuli

In subsequent blocks, stimuli were sampled from the 7 × 7 stimulus space to manipulate short-term speech input regularities across a canonical block that mirrored English acoustic regularities (orange squares, Fig. 1, middle panel) and an accented block that reversed the typical correlation between F0 contour and duration to create an artificial “accent” (orange squares, Fig. 1, right panel). Exposure stimuli comprised 80% of trials in these blocks.

### Test stimuli

Test stimuli (blue squares, Fig. 1) made up the remaining 20% of stimuli within the canonical and accented blocks. Test stimuli were constant across blocks and served to assess the degree to which listeners make use of duration to signal word emphasis in the context of the short-term regularities conveyed by the exposure stimuli that vary across blocks. Test stimuli had acoustically ambiguous F0 contour (level 50%) and distinct duration (level 33%, level 67%). Test stimuli only varied in the secondary dimension, duration, because our theory did not make specific predictions about changes in the primary dimension, F0 and were randomly interspersed with Exposure stimuli in the canonical and accented blocks.

### Procedure

Participants were seated in front of a computer monitor in a sound-attenuated booth. Each trial began with a looming checkerboard circle in the center of the monitor. When participants had fixated on the checkerboard for one second, a stimulus phrase “study music” (Fig. 1) was presented diotically over headphones (Beyer DT-150) and then the response options appeared on the screen. Participants were instructed to press either the “z” or “m” key on the keyboard, associated with the spatial position of the response labels, to indicate whether they heard “STUDY music” or “study MUSIC.” The key press triggered the next trial.

Participants experienced the baseline, canonical, and accented conditions as three blocks, always presented in the same order. The only difference between blocks was the sampling of stimuli. The task remained constant. Trials were presented across blocks without breaks or any other overt demarcation so that block structure was implicit and unknown to participants. The baseline block consisted of 200 trials (25 stimuli × 8 presentations; grey, Fig. 1), the canonical block consisted of 80 canonical exposure trials (10 stimuli × 8 presentations; orange, Fig. 1, middle panel) and 16 interspersed test trials (2 stimuli × 8 presentations; blue, Fig. 1), and the accented block consisted of 80 accented exposure trials (orange, Fig. 1, right panel) and 16 interspersed test trials (blue, Fig. 1). The entire session was completed in approximately 25 minutes. The experiment was delivered with E-Prime experiment software (Psychology Software Tools, Inc.).

### Analyses

F0 contour and duration perceptual weights for the baseline trials were calculated by estimating a logistic regression for each subject, with F0 level (2 to 6) and duration level (2 to 6) predicting the binary response (*STUDY music* vs *study MUSIC*). The coefficients for F0 contour and duration were then combined by normalizing them such that they summed to one (Holt & Lotto, 2006; Idemaru et al., 2012; Jasmin, Dick, Holt, & Tierney, 2020a), resulting in a normalized perceptual weight that ranged between 0 and 1, with values closer to 1 indicating greater reliance on F0 contour than duration, values closer to 0 indicating the reverse, and 0.5 indicating equal reliance. The mean normalized perceptual weights were compared across subjects against a value of 0.5 with a one-sample *t* test.

Performance on the exposure trials in the canonical and accented blocks was assessed for accuracy as proportion correct (defined according to the “dominant,” heavily perceptually weighted, dimension from the baseline weights). To analyze effects of canonical and accented exposure on categorization of test stimuli, the trial-wise data for all participants were entered into a mixed effects logistic regression using *lme4*’s *glmer* function (Bates et al., 2015) with “family=binomial,” and response (*STUDY music* vs. *study MUSIC*) predicted by the exposure type (canonical vs. accented), duration (longer *STUDY* vs. longer *MUSIC*), and their interaction, as well as *P*articipant as a random intercept, using R (R Core Team, 2021). The effect of the interaction term was calculated by comparing this full model with a null model (without the interaction) using R’s *anova* function. Bonferroni-corrected pairwise tests were conducted with the pairs function in the *lsmeans* package in R, which calculates and contrasts “estimated marginal means” for different factor combinations in mixed and other linear models (Lenth, 2016; Searle et al., 1980).

## Results

### Baseline categorization

**Fig. 2:**
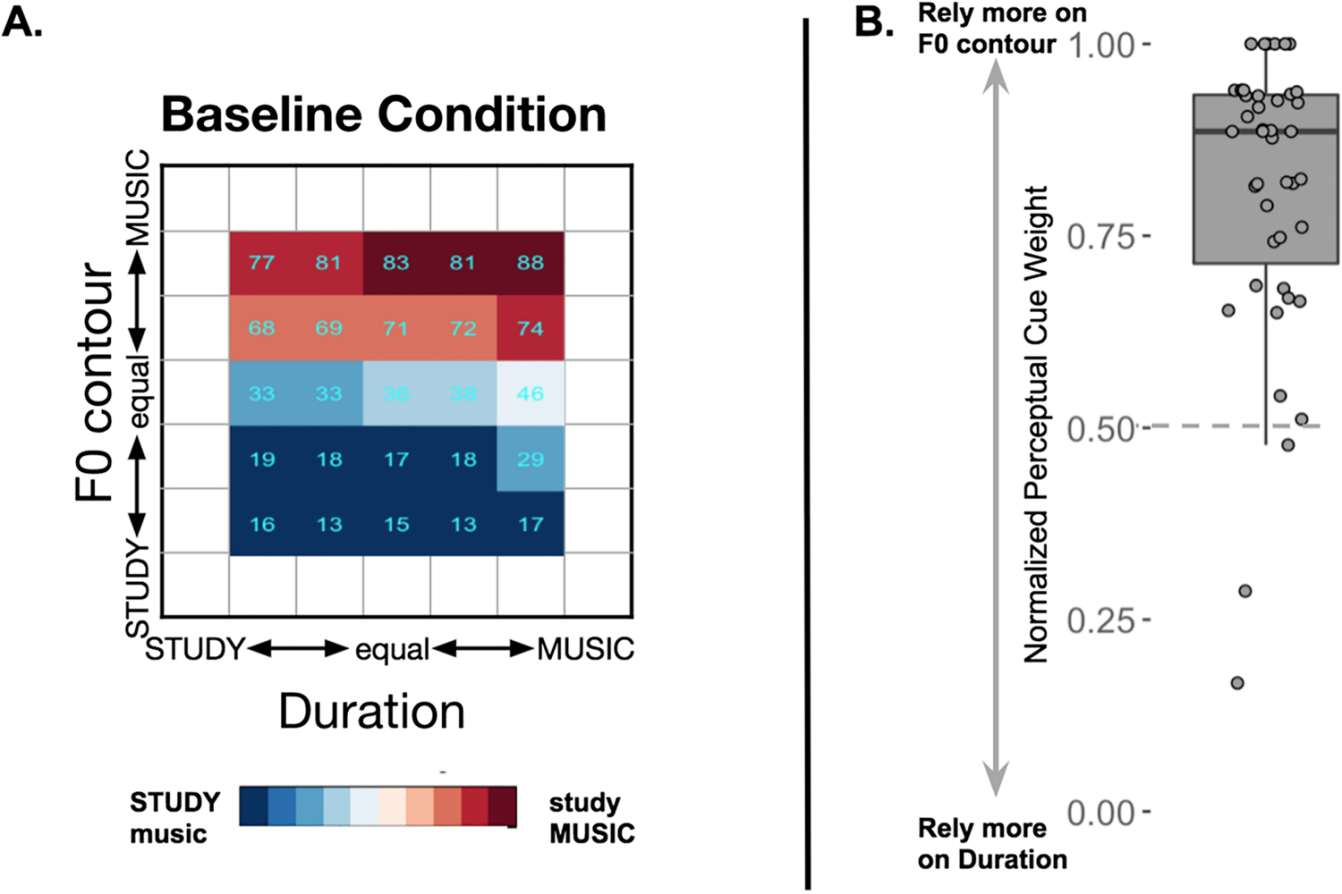
Results from the baseline condition. **a** Mean percentage categorization responses for each of the baseline stimuli. Blue indicates that participants tended to perceive emphasis as “*STUDY music*,” whereas red indicates that they perceived emphasis as “*study MUSIC.*” **b** Normalized perceptual weights for each participant. Most participants relied more on the F0 contour dimension than the duration dimension to judge emphasis. (Color figure online)

Figure 2 illustrates average categorization responses for the baseline block in which F0 contour and duration varied orthogonally across stimuli. Participants tended to rely more on F0 contour than duration to categorize the spoken phrase according to word emphasis, replicating the results of Jasmin, Dick, Holt, and Tierney (2020a), and confirming that F0 contour is a stronger cue to word emphasis than duration in English (normalized perceptual cue weight *M*_F0_ = 0.81±0.03), *t*(42) = 14.82, *p* < .001 (higher values indicate greater F0 contour reliance).

### Categorization of exposure stimuli

Responses to the unambiguous exposure stimuli were examined to ensure that participants were using F0 (the primary dimension signalling word emphasis) to make their judgments in the canonical and accented blocks (orange squares in Fig. 1). The mean percentage of correct responses, defined according to F0 contour, was high during the canonical block (*M* = 88.1 ± 12.1) as well as during the accented block (*M* = 81.9 ± 17.2).

### Categorization of test stimuli

**Fig. 3:**
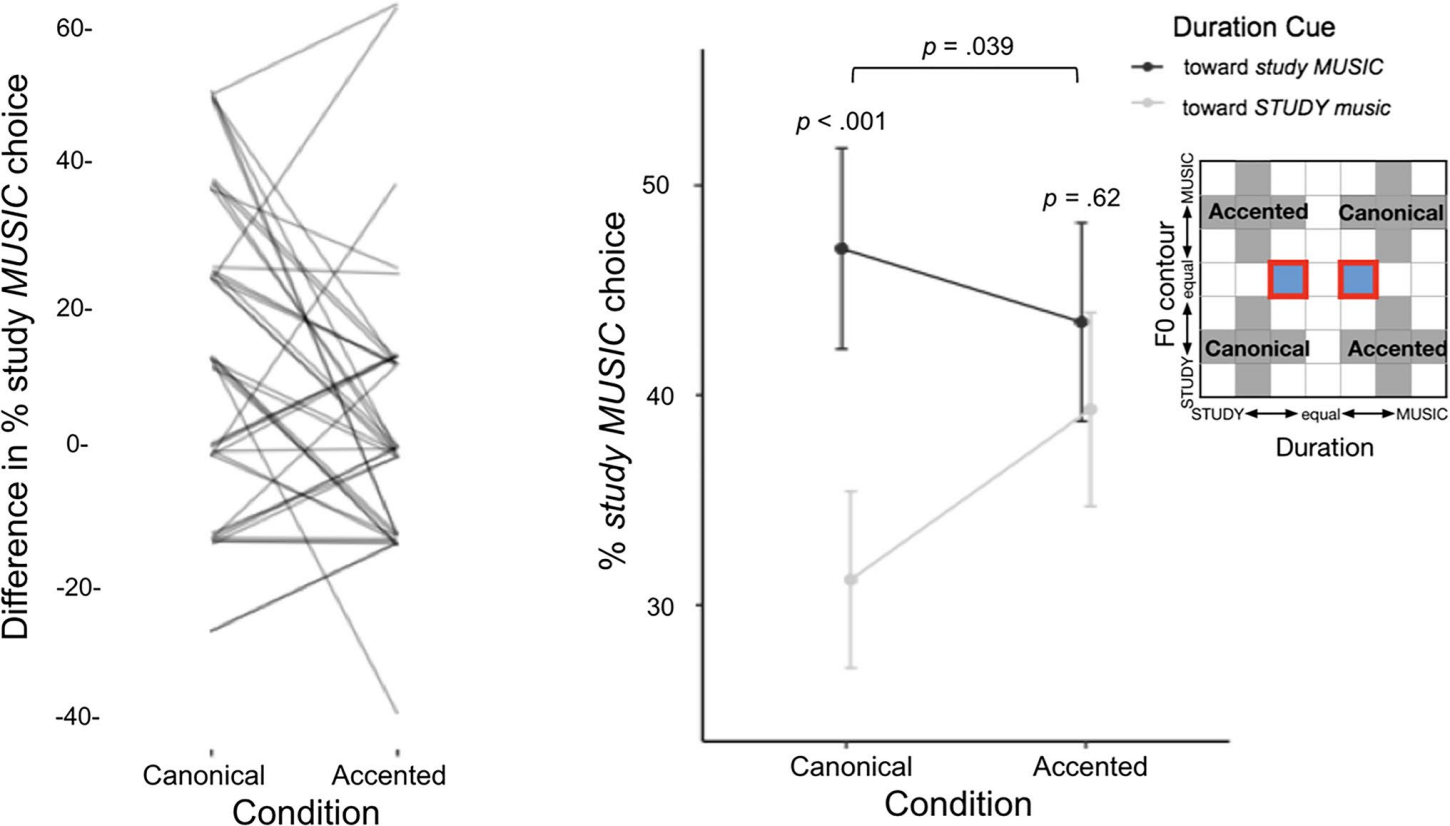
Test stimulus categorization in the context of canonical and reversed regularities. Suprasegmental categorization behavior in the context of exposure to canonical and accented statistical co-occurrence of F0 contour and duration dimensions. When short-term regularity aligned with long-term English regularities in the canonical block, duration differentially signalled word emphasis as *STUDY music* versus *study MUSIC*. Nonetheless, categorization of the same stimuli differed when short-term regularities departed from English in the accented block; participants no longer relied upon the duration dimension in word emphasis judgments. The left panel shows subject-level data: the difference in percent of *study MUSIC* responses across the test trials (blue squares in Fig. 1) for the canonical versus accented blocks. The right panel shows the mean percentage of responses categorized as *study MUSIC* for each test stimulus individually, and standard errors. Inferential statistics are the results of the mixed model analysis reported in the main text. (Color figure online)

Test stimuli served as the primary measure of whether short-term speech input regularities impact perception of word emphasis. Recall that test stimuli possessed an acoustically ambiguous F0 contour, thereby neutralizing the acoustic dimension most listeners rely upon to make word emphasis judgements (Fig. 2). Thus, categorization of test stimuli provides a measure of the extent to which listeners rely on duration to judge word emphasis, and whether the perceptual weight of duration is modulated across manipulations in short-term speech regularities experienced across exposure stimuli in the canonical and accented blocks. Figure 3 illustrates these results.

As predicted, categorization of the test trials differed as a function of the short-term speech input regularities experienced across the canonical and accented blocks (comparison of the full model including interaction of block and test stimulus duration term with null model omitting the interaction) χ^2^(1, 5) = 4.27, *p* = .039. Pairwise post hoc tests indicated that in the context of canonical short-term regularities in speech input conveyed by the exposure trials, duration influenced categorization when F0 contour was ambiguous, with longer word duration indicating emphasis (*OR* = 1.95, Cohen’s *d =* 0.37, Z = 3.90, *p* < .001). However, upon introduction of the artificial “accent” that reversed the relationship between F0 contour and duration relative to canonical English patterns, listeners’ reliance on duration to signal word emphasis rapidly shifted. In the context of exposure to the accented short-term regularity conveyed by exposure trials the perceptual weight of durational information dramatically decreased, to the point that there was no significant difference in participants’ word emphasis judgements as a function of duration (*OR* = 1.19, Cohen’s *d =* 0.10, Z = 1.02, *p* = .62).

## Discussion

While it had been well-established that the relative weighting of acoustic dimensions for segmental speech categorization can shift rapidly according to the listening context, short-term shifts in relative dimensional weighting of cues for suprasegmental perception had not yet been demonstrated. In the present work, we exposed listeners to an artificial “accent” in which the typical co-occurrence of F0 contour and duration for word emphasis in English was reversed. We found that the perceptual weight of duration sharply decreased in response to this shift in context, and we therefore conclude that perceptual cue weights for word emphasis are malleable, responding dynamically to statistical properties of the speech input.

These results are consistent with an account in which evidence accumulated across multiple acoustic dimensions (with greater weighting for certain “primary” dimensions) leads to activation of discrete multidimensional word emphasis categories. Category activation may then generate error signals due to mismatch between expected and actual values along acoustic dimensions (e.g., a word perceived as emphasized that is nonetheless short in duration). These error signals would then lead to adjustment of the effectiveness of input dimensions in subsequently signalling categories (Guediche et al., 2014; Idemaru & Holt, 2011; R. Liu & Holt, 2015; Wu & Holt, 2022).

It remains an open question whether other prosodic features are perceived as discrete multidimensional categories. Intonational phrase boundaries, for example, are accompanied acoustically by lengthening of the syllable just before the boundary, increased pause duration, and sudden changes in pitch (Choi et al., 2005; Cumming, 2010). Listeners integrate information across acoustic dimensions when interpreting the location of an intonational boundary (Beach, 1991; de Pijper & Sanderman, 1994; Streeter, 1978), and English speakers place greater weight on the durational than the pitch cues (Jasmin et al., 2021). Prior evidence regarding categorical perception of phrase boundaries is mixed: Some researchers have reported a discrimination peak aligned with a category boundary (Remijsen & van Heuven, 1999; Saindon et al., 2017a, b; Schneider & Lintfert, 2003), whereas other researchers reported finding no discrimination peak (Falé & Faria, 2006). Future work could investigate the existence of discrete multidimensional categories for phrase boundaries using a paradigm similar to that used in the current paper. If discrete multidimensional phrase boundary categories exist, we would predict that creation of an artificial accent in which pitch versus durational information supported contrasting phrase boundary interpretations would lead to short-term decreases in pitch weighting. If phrase boundaries are not perceived categorically, we would predict no short-term changes in weighting after exposure to the accent.

In this study, we examined a single context manipulation (“canonical” speech for which F0 and duration cues covaried normally and “accented” speech for which F0 and duration cues were opposite of the typical covariation pattern). We also presented only a single spoken token (“study music”) spoken by a single talker. It remains to be seen, therefore, whether short-term changes in word emphasis cue weights generalize to other suprasegmental features, other specific examples of word emphasis, or other speech from other talkers, who may use prosody variably (Peppé et al., 2000). Research on dimension-based statistical learning here has observed that some generalization takes place, but to different extents depending on speaker and linguistic contexts (Idemaru & Holt, 2014; Lehet & Holt, 2020; R. Liu & Holt, 2015; Zhang & Holt, 2018). For segmental speech perception, it has been shown that learning of an artificial accent generalizes across linguistic contexts (e.g., to lists of words/nonwords; Idemaru & Holt, 2020; Lehet & Holt, 2020; Zhang et al., 2021) and across voices (R. Liu & Holt, 2015; Zhang & Holt, 2018), but the degree of down-weighting tends to be lesser in contexts not directly experienced by listeners. There is also evidence that speaker information cued vocally or visually can be used to guide speaker-specific dimension-based statistical learning across phonetic categories, supporting simultaneous tracking of multiple input regularities (Zhang & Holt, 2018). Based on this prior evidence from research on segmental categorization, we predict that word emphasis down-weighting will generalize across voices but do not have a strong prediction regarding whether down-weighting will generalize to other suprasegmental features (e.g., from word emphasis to phrase boundaries). To the extent that short-term input regularities across acoustic dimensions are effective in activating word emphasis categories even as they deviate from long-term expectations of correlations among input dimensions, we would anticipate reweighting and modest generalization. In fact, as has been the case in studies of segmental categories (Idemaru & Holt, 2014), successes and failures of the generalization of dimension-based statistical learning can inform the nature of underlying category representations.

Here, we find that when covariation between F0 and duration is opposite that of the typical relationship in English, listeners down-weight duration but continue to rely on F0, due to F0 being a statistically more reliable cue to the presence of word emphasis in English. However, F0’s dominance as a cue to word emphasis may not be universal across all English listeners, but instead may vary as a function of the experienced overlap in the distribution of cues associated with emphasized versus not-emphasized words (Holt & Lotto, 2006; Toscano & McMurray, 2010). For example, individuals with congenital amusia, who have difficulty perceiving and remembering pitch in both musical and speech stimuli, weight F0 and duration roughly equally in a word emphasis categorization task like the one studied here (Jasmin, Dick, Holt, & Tierney, 2020a). We predict, therefore, that individuals with amusia would not down-weight duration when exposed to “accented” speech in which pitch and duration suggest conflicting interpretations regarding word emphasis. F0 may also not be the most reliable cue to word emphasis for all speakers of English. When conveying the distinction between question and statements, for example, although adults and older children primarily rely on F0 with duration and intensity playing a secondary role, younger children primarily rely on duration (Patel & Grigos, 2006). This may be due to a lack of control over F0 in younger children, which could lead F0 to be deemphasized as a cue to other suprasegmental features as well, including word emphasis. If so, we predict that listeners would down-weight F0, rather than duration, when exposed to “accented” stimuli drawn from the speech of young children in which pitch and duration suggest conflicting interpretations regarding word emphasis.

Our results suggest that perceptual weights for word emphasis are not fixed. Instead, they continually adjust in response to short-term speech input regularities. Future work could investigate whether suprasegmental dimensional weighting reflects the relative utility of different cues in particular listening environments, or as a function of task (Holt & Lotto, 2006). For example, prior work in segmental perception shows that weighting of an F0-based cue to voicing (the F0 of the vowel following the consonant) is increased when speech is presented in masking noise, while the duration-based cue (VOT) is down-weighted (Holt et al., 2018; Winn et al., 2013; Wu & Holt, 2022). Similarly, distinct contexts and task demands are likely to impact the relative effectiveness of multidimensional acoustic information signaling suprasegmental categories, as they do for segmental categories.

The size of the steps between F0 levels was large compared with the size of the steps between duration levels, relative to average discrimination thresholds in the general population (Kidd et al., 2007). It is plausible, therefore, that the F0 levels were easier to discriminate, which could be one reason why F0 tends to be the primary dimension across listeners. However, we would argue that this difference cannot drive our primary finding that listeners down-weight duration during exposure to the Accented distribution. This is because, as a group, the participants responded differently to the test stimuli (which differed only in duration) in the context of exposure to the canonical distribution.

Our results suggest that dimensional weighting during perception of word emphasis is a dynamic process, in that relative weighting can change over the time scale of just a few minutes. What neural mechanism might make possible these rapid changes in how perceptual information is integrated? One possibility is that short-term modulations in neural functional connectivity between perceptual regions that process a given acoustic dimension and regions associated with language processing drive changes in dimensional weighting. We recently presented evidence suggesting that functional connectivity patterns may underlie relative perceptual weighting of acoustic dimensions during suprasegmental speech perception as well (Jasmin, Sun, & Tierney, 2020b). We found that when participants underwent fMRI scanning while performing an intonational phrase boundary perception task, connectivity between pitch-sensitive areas in the insula and superior temporal gyrus and left prefrontal language-related regions was weakened in participants with amusia, who down-weighted pitch information during suprasegmental categorization, relative to control participants. This connectivity pattern, however, could reflect intrinsic differences between amusics and controls rather than perceptual weighting. The hypothesis that dimensional weighting is linked to changes in the degree of correlated activity between task-related brain areas could be more stringently tested using the word emphasis dimensional weighting shift paradigm presented in the current paper, by inducing shifts in cue weighting driven by contextual changes in the correlations between dimensions and examining the effects on functional connectivity. There is also important work to be done to understand which mental representations are impacted, and how distributions of speech input interact with a system tuned to expect specific regularities characteristic of a language community.

It is also possible that dimension-based statistical learning of cues to word emphasis may extend to production. In a study on segmental speech, exposure to a reverse (“accented”) correlation between F0 and VOT led to down-weighting of F0 in perceptual category decisions and also diminished participants’ own use of F0 in their speech productions (Lehet & Holt, 2017). Further work could investigate whether the down-weighting of duration observed here during word emphasis perception also manifests in speech production acoustics, which would suggest that word emphasis categories activated during perception are shared with production.

Our theoretical model of the relationship between category activation and changes in perceptual cue weighting is that discrepancies between secondary dimension values normally associated with an activated category and those actually perceived lead listeners to temporally down-weight the secondary dimension. Our primary hypothesis, therefore, only referred to secondary dimensions, and so we did not include test trials in which duration was ambiguous and F0 varied. One limitation of this approach, though, is that it leaves open the question of whether changes in secondary cue weighting have subsequent effects on primary cue weighting. In other words: when listeners down-weight duration as a cue to word emphasis, do they up-weight F0 to compensate? Future work could investigate this issue by including both F0-varying and duration-varying test trials.

In conclusion, we find that dimensional weights in prosodic speech perception are signalled by multiple acoustic dimensions whose perceptual weights are flexible rather than fixed: They rapidly change in response to alterations in the distributional characteristics of dimensional cues in the input. This suggests that prosodic speech perception involves combining information from multiple sources to perceive multidimensional prosodic categories.

## Supplementary Information

ESM 1 (DOCX 1953 kb)
