# Supplementary material for: Short-term perceptual reweighting in suprasegmental categorization
Source: Psychon Bull Rev. 2022 Aug 1;30(1):373–82. doi: 10.3758/s13423-022-02146-5 (PMC9971089; doi:10.3758/s13423-022-02146-5)
Supplement: Supplementary file 1 — (DOCX 1953 kb) [file 13423_2022_2146_MOESM1_ESM.docx]

# Online Supplemental Materials

**Stimulus creation: morphing procedure**

Recordings were made of a Standard Southern British English-speaking voice actor reading aloud two different sentences: “Dave likes to STUDY music, but he doesn’t like to PLAY music” (Recording A) and “Dave likes to study MUSIC, but he doesn’t like to study HISTORY” (Recording B). The first five words of each recording were extracted; these recordings were identical lexically but differed in the placement of contrastive focus, i.e. on STUDY in the first recording and on MUSIC in the second recording. The speech morphing software STRAIGHT (Kawahara & Irino, 2005) was then used to morph Recording A and Recording B recordings onto one another using the standard procedure: the F0 was extracted from voiced segments of the two utterances; next, aperiodic aspects of the signal were identified and analyzed; then, the filter characteristics of the signal were calculated. Finally, the two “morphing substrates” (speech from each recording decomposed into F0, aperiodic aspects and filter characteristics) were manually time aligned by marking corresponding ‘anchor points’ in both recordings, such as the onsets of energy changes (e.g. bursts), so that morphs reflect the temporal characteristics of the two initial recordings, varying in the extent to which acoustic cues imply the existence of a phrase boundary either at the middle or at the end of the phrase. Pitch and duration were set to vary across seven morphing equal morphing steps, expressed as percentages, from 0% (identical to the acoustic pattern of Recording A to 100% (identical to the pattern for Recording B).


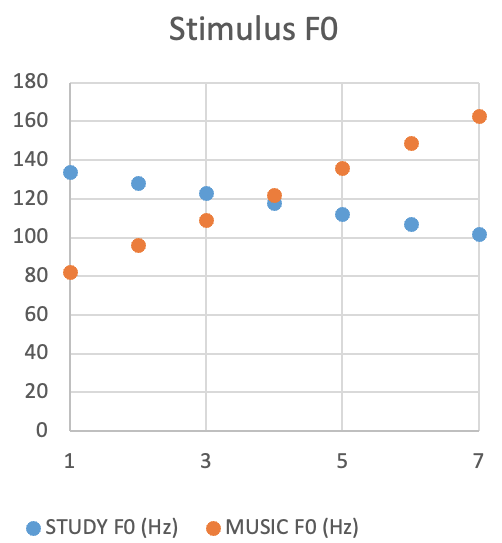


Supplemental Figure 1: Stimulus F0 values measured at the nucleus of the stressed syllable of “STUH” in STUDY and “MEW” in MUSIC, across the 7 morphing levels.


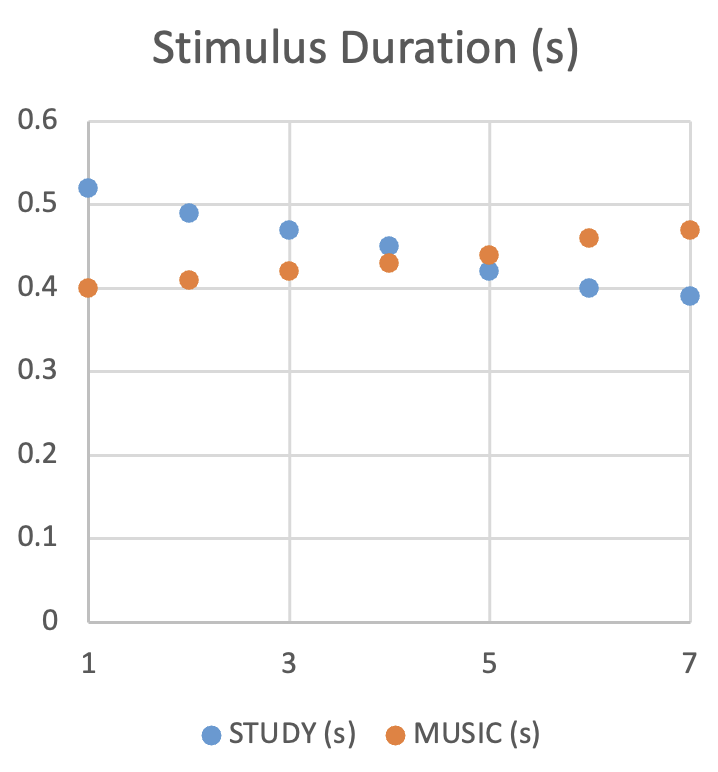


Supplemental Figure 2: Durations of the words STUDY and MUSIC across the 7 morphing levels.


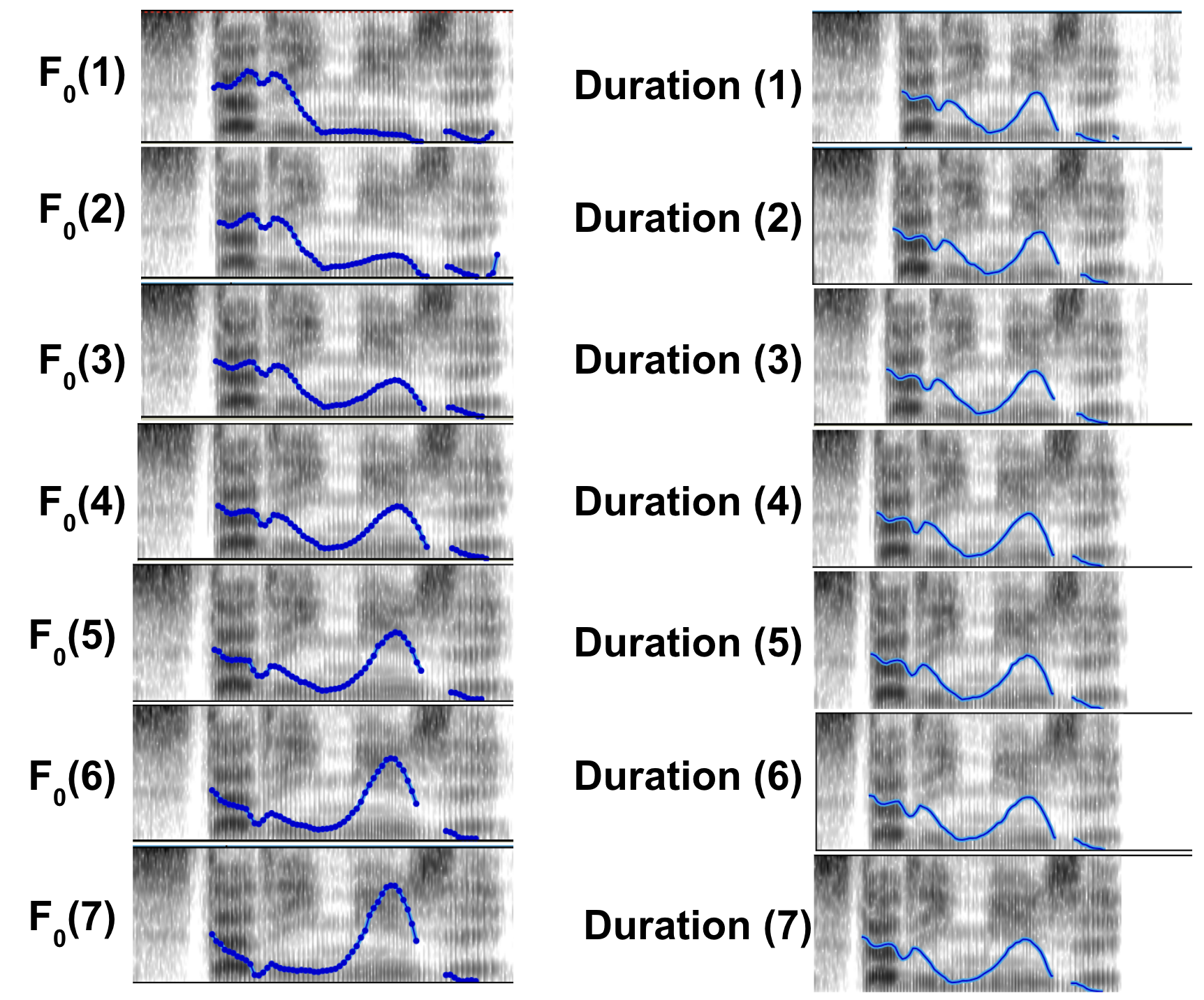


Supplemental Figure 3: F0 and Duration patterns across the stimulus levels. Left column displays the F0 contours (with the duration pattern held constant). Right column displays the duration patterns (with F0 held constant).
